# Modelling the Full-Length Inactive PKC-δ Structure to Explore Regulatory Accessibility and Selective Targeting Opportunities

**DOI:** 10.3390/ph18111760

**Published:** 2025-11-18

**Authors:** Rasha Khader, Lodewijk V. Dekker

**Affiliations:** 1 School of Pharmacy, Biodiscovery Institute, University of Nottingham, Nottingham NG7 2RD, UK; 2 Department of Clinical Pharmacy, Faculty of Pharmacy, Jordan University of Science and Technology, Irbid 22110, Jordan

**Keywords:** protein kinase C-δ, comparative modelling, ligand docking, kinase regulation, breast cancer

## Abstract

**Background/Objectives:** Protein kinase C-δ (PKC-δ) is a pivotal regulator of cellular signalling, and its dysregulation contributes to oncogenesis. While certain isolated PKC-δ domains have been crystallised, the full-length architecture and interdomain interactions remain largely unresolved, limiting mechanistic insight and the design of selective modulators. We aimed to define the full-length, inactive conformation of PKC-δ and identify accessible, functionally relevant binding sites for ligand discovery. **Methods:** We generated a consensus structural model of full-length inactive PKC-δ using multi-template comparative modelling guided by established inactivity markers. Molecular docking was used to predict ligands targeting the C2 domain, which were subsequently validated in breast cancer cell models, including wild-type and C2 domain-overexpressing lines. **Results:** Analysis of the model revealed the architecture of the C2/V5 interdomain space, providing a structural rationale for regulation of the nuclear localisation signal (NLS). Docking identified two ligand classes: ligand 1 engaged a C2 domain surface oriented toward the C2/V5 pocket, while ligand 2 targeted the C2 domain phosphotyrosine-binding domain (PTD). Experimental validation in breast cancer cell models demonstrated that both ligands reduced cell viability; ligand 1 showed enhanced effects in C2-overexpressing cells, consistent with predicted accessibility, whereas ligand 2 partially counteracted the C2 domain-induced viability phenotype, likely via interference with PTD-mediated interactions. **Conclusions:** Full-length structural context is essential for identifying accessible, functionally relevant binding sites and understanding context-dependent kinase regulation. Integrating computational modelling with phenotypic validation establishes a framework for selective PKC-δ modulation, offering insights to guide ligand discovery, improve isoform selectivity, and inform strategies to mitigate kinase inhibitor resistance in precision oncology.

## 1. Introduction

Protein kinase C-δ (PKC-δ) is a member of the PKC family, a subgroup of the AGC kinase superfamily that regulates diverse cellular processes, including proliferation, differentiation, and apoptosis [1,2]. The PKC family is classified into classical, novel, and atypical isoforms based on their activation requirements and domain composition [3]. In general, PKCs transition between a closed, auto-inhibited conformation and an open, catalytically active state, a process regulated by second messengers, (auto)phosphorylation, or protein interactions [3,4].

PKC-δ, a member of the novel subclass, is primarily regulated by diacylglycerol rather than calcium [3]. It also undergoes regulation through tyrosine phosphorylation, which controls its subcellular localisation and activation state [5,6,7,8]. Importantly, in cancer, PKC-δ exhibits dual, context-dependent functionality, exerting either pro- or anti-apoptotic effects depending on the cellular environment, highlighting ongoing debates about its precise role in cancer progression [1,2]. For example, phosphorylation at Tyr64 exposes a nuclear localisation signal (NLS) within the V5 domain, enabling nuclear import and promoting apoptosis [9], while phosphorylation at Tyr334 influences caspase-mediated cleavage and generation of a constitutively active catalytic fragment [5,10]. Conversely, activating the C1 domain via increased intracellular diacylglycerol or phorbol esters induces membrane binding and enhances cell survival [11]. These examples demonstrate that PKC-δ function is both domain- and context-specific, emphasising that selective modulation rather than global inhibition is required to achieve desired cellular outcomes.

Structurally, PKC-δ comprises conserved regulatory (C1–C2) and catalytic (C3–C4) domains interspersed with variable regions (V1–V5), forming a modular, conformationally dynamic architecture [3]. While individual domains such as C1 and C2 have been crystallised (e.g., PDB: 1BDY, 1YRK, 2YUU) [4,5,8], the spatial arrangement of these domains in the full-length protein remains unresolved. This lack of structural information limits mechanistic understanding of how interdomain interactions govern autoinhibition and functional output, and it complicates strategies to selectively modulate regulatory or catalytic modules.

Computational multi-template modelling offers a means to reconstruct full-length PKC-δ architectures by integrating homologous templates guided by experimentally supported inactivity markers [12,13]. In AGC kinases, these markers, such as pseudosubstrate engagement and C-terminal domain clamping, offer biologically meaningful constraints for model building [6,7,14,15]. Defining the full-length architecture enables mechanistic insight into how transient interdomain contacts regulate domain accessibility and functional output, providing a framework to guide selective modulation of regulatory or catalytic domains in a context-dependent manner.

We hypothesise that previously unresolved interdomain contacts control catalytic accessibility and regulatory module function, revealing structurally accessible regions that can be targeted to selectively enhance or suppress PKC-δ functions. Our consensus structural model captures the dynamic intramolecular architecture underlying kinase autoinhibition and identifies accessible regions within regulatory and catalytic domains, offering opportunities to manipulate specific activation pathways to modulate apoptosis, cell cycle progression, or survival. These structural insights provide a foundation for rational strategies to selectively modulate PKC-δ function in cancer, potentially informing therapeutic intervention and overcoming domain-specific resistance mechanisms.

## 2. Results

### 2.1. Consensus Structural Model of the Kinase

**Figure 1:**
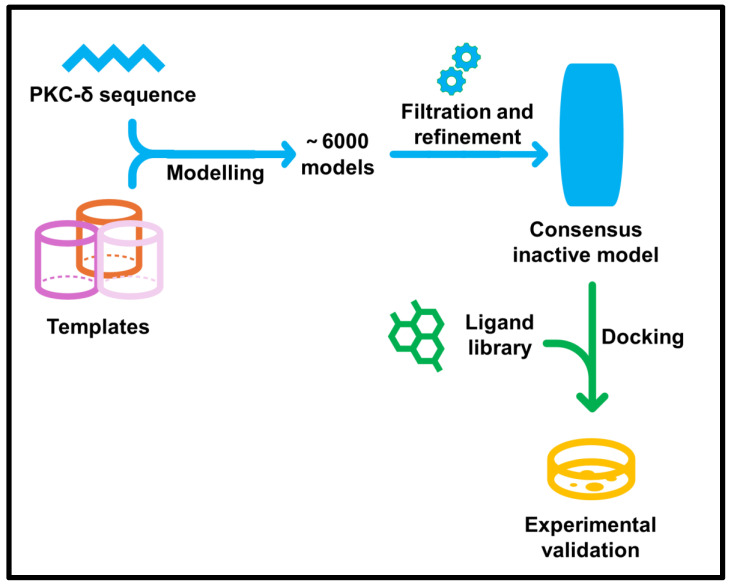
Workflow for structural modelling and validation of protein kinase C-δ (PKC-δ). The PKC-δ sequence was aligned to multiple templates, and ~6000 models were generated by comparative modelling. After iterative filtration, refinement, and re-modelling, a consensus inactive model was selected. The model was then validated through virtual high-throughput docking of a ligand library, followed by experimental confirmation of top-ranked ligand activity in cell-based assays.

Comparative modelling of the inactive full-length kinase required balancing multiple domain templates (Supplementary Table S1). Template order strongly influenced model quality: some orders produced plausible domain packing, whereas others yielded intercalated or misfolded domains. Iterative multi-template modelling (~6000 models in total), followed by visual inspection and stereochemical scoring, resulted in several hundred structurally consistent candidates (Figure 1; detailed workflow in Supplementary Materials Section S2). From these, a consensus model was assembled by integrating the most favourable features across independent trials. This systematic approach ensured that the final model was not the product of a single template combination but a consensus representation of the most recurrent and stereochemically stable inactive conformations.

**Figure 2:**
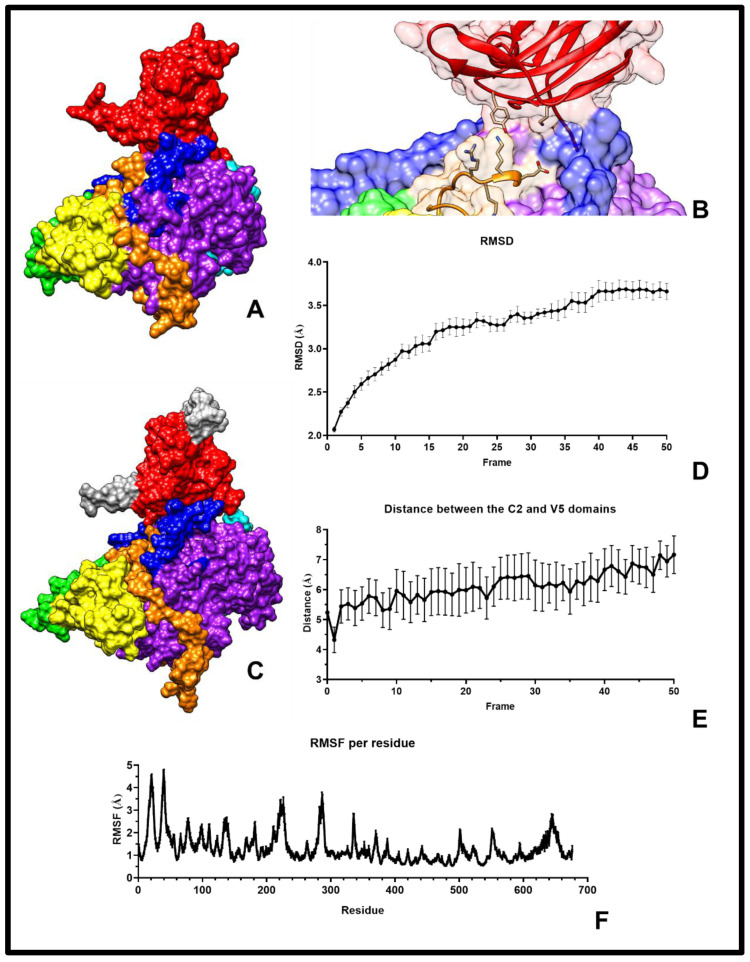
Model and validation. (**A**) The consensus model with coloured domains: C2 in red, pseudosubstrate in cyan, C1a in yellow, C1b in green, V3 in blue, C3 and C4 in purple, and V5 in orange. (**B**) Close-up of the C2 and V5 domain arrangement. The C2 domain (red) residues Tyr64 and Lys48, and the V5 segment (orange) residues E^612^KRR^615^ are shown for reference. (**C**) A representative model after a 5 ns simulation (last frame), showing that the relative positioning of the C2 and V5 domains is preserved. Domains are coloured as in panel (**A**). (**D**) Average RMSD per frame for the 5 ns simulations (10 replicates, 10 frames/ns) calculated using VMD [17]. (**E**) Fluctuation in distance between the C2 and V5 domains across frames during the 5 ns simulations. (**F**) RMSF per residue, with highly fluctuating regions located at the N-terminal, highlighted in silver in panel (**C**). Error bars in panels (**D**–**F**) represent mean ± SEM of N = 10 independent molecular dynamic simulation replicates, each initiated from the same model structure with randomised initial velocities.

To assess the biological plausibility of the inactive conformation, we examined well-established inactivity markers in the AGC kinases. In the consensus model (Figure 2A), the pseudosubstrate residues Thr141, Arg144, and Arg145 occupy the substrate-binding site, shielded by an acidic helix (H^549^GDDEDE^555^) [16]; the V5 domain is clamped by the C1b subdomain [6]; the membrane-binding loop of the C1b subdomain (V^255^LGWK^256^) is oriented toward the catalytic domain [6]; and the conserved NF^633^D motif forms a helix positioned outside the ATP-binding site [6]. These features are consistent with inactive conformations reported in related kinases, supporting the use of the model for subsequent structural and ligand analyses, without implying it is a definitive experimental structure.

Notably, in every correctly folded model (>1000), the C2 domain consistently juxtaposed against the V5 domain NLS (E^612^KRRLEPPFRPKVK^625^), with the space prominently involving the C2 domain Tyr64 at the centre (Figure 2B). This arrangement was not enforced during modelling but nonetheless emerged reproducibly, suggesting a previously unrecognised structural interdomain space. Given its recurrence across independent models, this C2/V5 interdomain space represents a novel structural feature of PKC-δ architecture and became a focal point for subsequent analyses.

However, the C2 domain was not the only contributor to masking the V5 domain NLS. The V3 domain and the C1b subdomain also participated in occluding the NLS (Supplementary Figure S3). Whilst we enforced masking of the C1b subdomain (based on the known PKC-βII template; PDB: 3PFQ) and partially enforced the V3 domain mask (PDB: 1YRK), we did not enforce the C2 domain masking of the V5 domain NLS at any stage of the modelling.

### 2.2. Model Refinement and Validation

Iterative refinement progressively improved stereochemical quality, yielding clashscores of 0, Cβ deviations of 1.58%, 96.4% of residues in Ramachandran-favoured regions, and a MolProbity score of 0.94 (100th percentile; Supplementary Table S2). These metrics indicate that the consensus model is stereochemically robust and free of major structural anomalies.

To assess local stability and domain arrangement, short all-atom molecular dynamics (MD) simulations were performed (10 × 5 ns; Figure 2C–F). The simulations confirmed that the relative positioning of key domains was preserved, with the C2 domain consistently overlaying V5 and the catalytic core (Figure 2C), maintaining its structural integrity. No significant unfolding or domain misorientation was observed, suggesting that the consensus model supports the proposed inactive conformation of full-length PKC-δ.

Overall, these results support the reliability of the consensus model for downstream structural analyses. In particular, virtual ligand docking was used to validate the accessibility and plausibility of predicted regulatory pockets at the interdomain space.

### 2.3. Ligand Screening and Pocket Specificity in the C2 Domain

**Figure 3:**
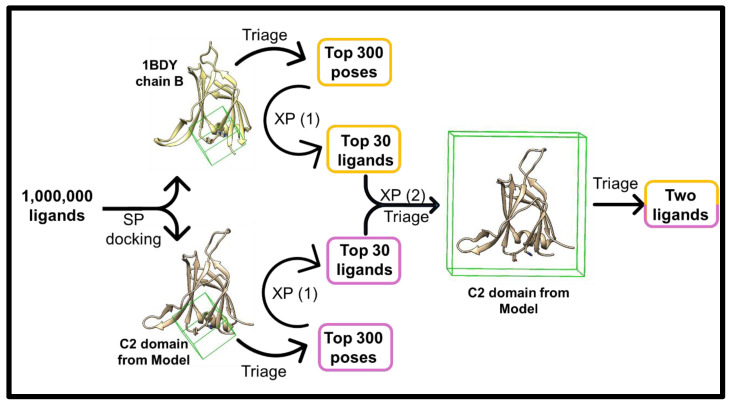
Docking workflow pipeline. Ligands were initially docked using Glide SP [18] into a defined region (green grid) in the C2 domain, once using the crystal structure (PDB: 1BDY, chain B) and once using the C2 domain isolated from the full-length model. The top 300 poses were triaged based on docking score, size, binding mode, and commercial availability, then redocked using Glide XP. After a second triage, the top 30 ligands from each run were docked into the full C2 domain from the model (XP (2) run), with the grid encompassing the entire domain to allow ligand exploration of any pocket. Final triage prioritised commercial availability for experimental testing. The docking and triage process was performed as a single, comprehensive analysis. The final selected ligands represent the consensus from this singular workflow.

To evaluate druggability, we first isolated the C2 domain from our consensus model and, in parallel, used the crystal structure of the isolated C2 domain (1BDY-B). Approximately 1,000,000 drug-like compounds were screened using a tiered SP/XP docking funnel, with visual inspection of binding interactions and ligand size of the top poses. From each structure, the 30 most promising compounds were selected and redocked into the full C2 domain of our consensus model (XP (2) docking, Figure 3), where the grid encompassed the entire domain rather than a pre-defined cavity. This strategy yielded two final candidate ligands with distinct behaviours; both of which were the highest scoring in the XP (2) docking run, which were commercially available for immediate purchase at the time.

**Figure 4:**
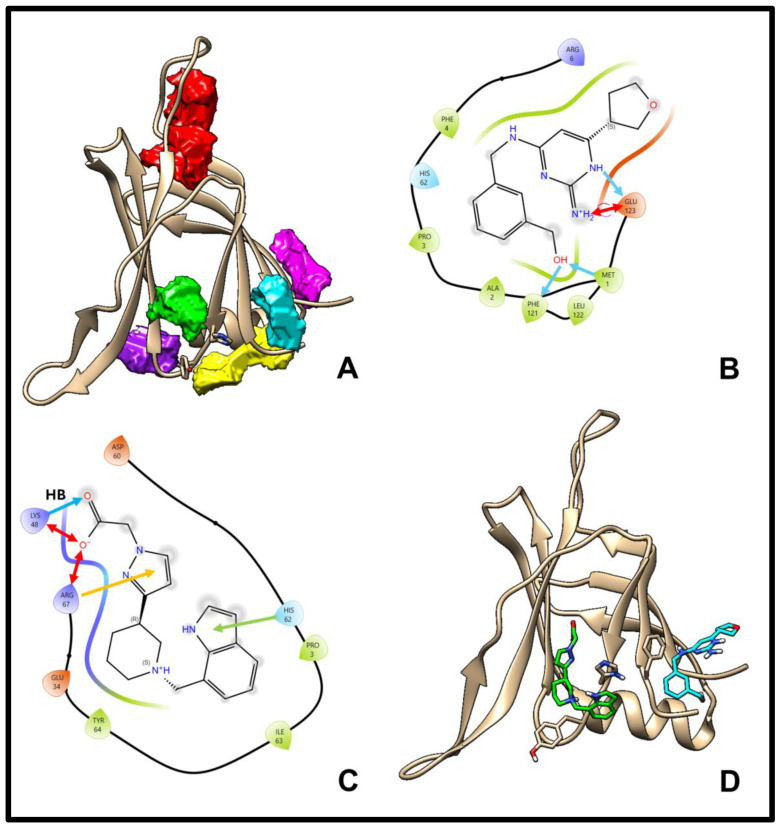
Druggable pockets and ligand interactions in the C2 domain. (**A**) Six druggable pockets were identified in the isolated C2 domain (model) using ProteinPlus DOGSiteScorer [19]. Three pockets (yellow, green, cyan) cluster around the C2/V5 interdomain space, with the red pocket ranked as the most druggable. (**B**,**C**) Maestro 2D interaction diagrams of (**B**) ligand 1 and (**C**) ligand 2. Arrows: blue = hydrogen bonds, red = salt bridges, orange = π-cation, green = π-π interactions. (**D**) 3D representation of ligand 1 (cyan) and ligand 2 (green) bound to the C2 domain after Glide XP (2) docking. Phe4, Tyr64, and His62 are shown for reference.

While six pockets across the entire C2 domain were identified as potentially druggable (Figure 4A), our primary goal was to assess whether the detected cavities corresponded to functionally relevant interdomain spaces, particularly the C2/V5 region (pockets green, yellow, and cyan, Figure 4A). Notably, the green pocket overlapped with the putative phosphotyrosine-recognition site of the C2 domain, defined by Lys48, His62, and Arg67, which is proposed to engage phosphorylated ligands through aromatic interactions [5]. For pocket metrics, see Supplementary Table S3.

Ligand 1 (Figure 4B) was not the top-ranking compound when docked into the specific pocket of the isolated C2 domain. However, it became the highest-ranking ligand after XP (2) docking into the isolated full C2 domain with no pre-defined pocket. After XP (2) docking run, ligand 1 changes its binding pocket from pocket yellow to pocket cyan (cyan ligand; Figure 4D). On the other hand, ligand 2 (Figure 4C; green ligand in Figure 4D) was neither the first-ranked compound when docked to the specific pocket in 1BDY-B nor the top-ranked ligand after XP (2) docking into the full C2 domain (it ranked second) and largely retained its original binding pocket (pocket green; Figure 4A). These pockets in the full-length model are positioned at the C2 surface facing the C2/V5 interdomain space, a placement not evident in the isolated C2 domain structure.

This divergence in pocket preference was mirrored in subsequent cell-based assays: ligand 1 produced effects consistent with perturbing the C2/V5 domain arrangement, whereas ligand 2 showed behaviour indicative of engagement with the phosphotyrosine-binding domain (PTD). These observations support the structural validity of the predicted interdomain interface and demonstrate that the model captures context-dependent ligand specificity. For further details on docking grids, binding mode and interactions, and ligand properties, see Supplementary Figure S4 and Tables S4 and S5.

### 2.4. Ligands Reduce Wild-Type Breast Cancer Cell Lines Viability

**Figure 5:**
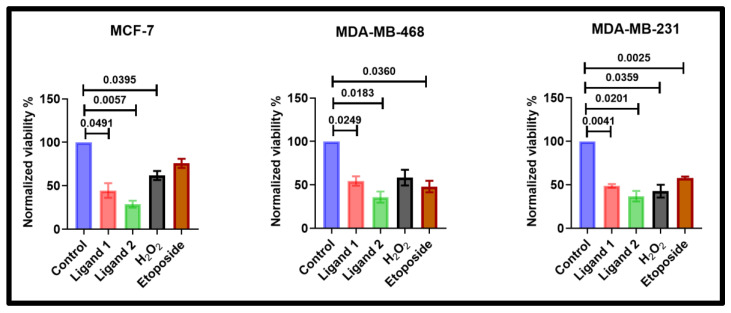
Effect of ligand treatment on viability of breast cancer cell lines. MCF-7, MDA-MB-468, and MDA-MB-231 cells were treated with 100 μM of each ligand for 48 h. Etoposide (100 μM) and H_2_O_2_ (100 nM) were included as positive controls. Cell viability was normalised to untreated controls (set as 100%). Statistical significance was assessed by one-way ANOVA followed by Dunnett’s multiple comparisons test. Bars represent mean ± SEM of N = 4 independent biological replicates, each is the average of two technical replicates.

In untransfected wild-type (WT) MCF-7, MDA-MB-468, and MDA-MB-231 cells, 48-h single-agent treatment with either ligand 1 or ligand 2 (100 μM) significantly (*p* < 0.05) reduced viability compared to untreated controls in all three cell lines (Figure 5). Etoposide (100 μM) and H_2_O_2_ (100 nM) served as positive controls, both being established activators of PKC-δ that induce phosphorylation at multiple tyrosine residues, including Tyr64 at the predicted C2/V5 interdomain space [9,10,20].

### 2.5. Comparative Effects of Ligands on Viability in δC2- and Vector-Transfected MCF-7 Cells

As previously established, MCF-7 cells stably overexpressing the isolated PKC-δ C2 domain (δC2 cells) exhibit reduced viability relative to Vector controls, due to increased apoptosis and G2/M cell cycle arrest [21]. These cells are also sensitised to H_2_O_2_ and etoposide treatments [21], demonstrating that the C2 domain can independently perturb cell survival. The δC2 and Vector cell lines used here were derived from this earlier work.

**Figure 6:**
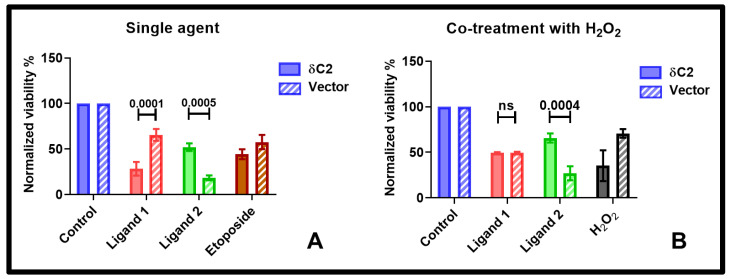
Effect of ligand treatment on viability of δC2-overexpressing MCF-7 cells. (**A**) δC2-overexpressing (δC2) and Vector control MCF-7 cells were treated with 100 μM of each ligand for 72 h. Etoposide (100 μM) was included as a positive control. (**B**) δC2 and Vector cells were co-treated with 100 μM of each ligand and 100 nM H_2_O_2_ for 72 h. Cell viability was normalised to untreated controls (set as 100%). Statistical significance was determined by two-way ANOVA followed by Tukey’s multiple comparisons test. Bars represent mean ± SEM of N = 3 independent biological replicates, each is the average of two technical replicates. ns = not significant.

Building on this, treatment of δC2 cells with ligand 1 (100 μM, 72 h) further reduced viability relative to Vector cells (28% in δC2 vs. 66% in Vector cells; Figure 6A). Interestingly, co-treatment of δC2 cells with H_2_O_2_ (100 nM, 72 h) and ligand 1 led to a “reset” effect, with viability increasing to 49% compared to untreated controls (δC2 + ligand 1 = 28%, δC2 + H_2_O_2_ = 35%, δC2 + H_2_O_2_ + ligand 1 = 49%; Figure 6B). This suggests that the cytotoxic effect of ligand 1 in δC2 cells is modulated by the phosphorylation state of its binding pocket.

In contrast, ligand 2 (100 μM, 72 h) attenuated the cytotoxic effects of C2 domain overexpression (Figure 6A). Co-treatment with H_2_O_2_ further enhanced this attenuation, consistent with the hypothesised phosphorylation-dependent modulation of PTD accessibility (Figure 6B).

To explore the relationship between predicted binding affinity, ligand/protein interactions, and cellular outcomes, we compared XP (2) docking scores of the two ligands with normalised cell viability in wild-type and δC2-overexpressing MCF-7 cells (Supplementary Tables S4 and S5). In wild-type cells, ligand 2 reduced viability more than ligand 1 despite a less favourable docking score, indicating that docking predictions alone do not fully capture cellular activity. Ligand 1 forms multiple hydrogen bonds and a salt bridge, whereas ligand 2 engages key residues through hydrogen bonds, salt bridges, and π-π/π-cation interactions (see Supplementary Table S5). In δC2-overexpressing cells, ligand 1 elicited stronger cytotoxicity, consistent with its more favourable docking score and bonding interactions, while ligand 2 produced weaker effects. These findings support the structural and functional relevance of the docking model, showing that predicted interaction patterns align more closely with experimental outcomes when the target C2 domain is overexpressed.

## 3. Discussion

Our study provides a comprehensive structural and functional characterisation of PKC-δ, combining comparative modelling, ligand docking, and cell-based validation to uncover new insights into kinase regulation. The consensus structural model of full-length PKC-δ incorporates well-established inactivity markers, including the pseudosubstrate occupying the substrate-binding site. [16], the V5 domain clamped by the C1b subdomain [6], the C1b membrane-binding loop oriented toward the catalytic domain [6], and the conserved NF^633^D motif positioned outside the ATP-binding site [6,22]. While not an experimental structure, the presence of these markers supports a biologically plausible inactive conformation.

Importantly, the full-length model enabled observation of interdomain arrangements, notably the C2/V5 interdomain spacing, which remained sufficiently consistent to prevent NLS exposure and nuclear translocation [23]. This supports our hypothesis that the C2/V5 arrangement maintains PKC-δ in an inactive, domain-clamped state. Additional domains, including V3 and C1b, also contribute to NLS masking, consistent with inactivity mechanisms observed in related PKC isoforms, such as PKC-βII and PKC-α [6,24,25]. Compared to AlphaFold predictions [26], which feature a detached, structurally unstable C2 domain floating away from the kinase core, our model integrates functional constraints and experimentally informed inactivity markers, providing a mechanistically interpretable framework rather than a purely statistical prediction.

While C2/V5 domain interactions have been reported in other PKC isoforms [24,25], our model uniquely positions Tyr64 at the interface, a residue specific to PKC-δ even relative to the most homologous isoform, PKC-θ [27]. This placement is consistent with prior functional data showing that Tyr64 phosphorylation exposes the V5 NLS [23,28,29,30,31], as the residue lies on the C2 face toward the C2/V5 interdomain space. Critically, these structural predictions are supported by experimental evidence: Adwan et al. demonstrated that a phosphomimetic Y64D mutation is sufficient to induce a lipid-independent active conformation, consistent with the domain separation and NLS exposure predicted by our model [28]. Moreover, the successful identification of functional ligands using both the modelled full-length and the crystallised isolated C2 domains highlights the value of a full-length model in revealing interdomain pockets that might not be apparent from isolated domains alone.

Short MD simulations (10 × 5 ns) consistently preserved interdomain spacing and domain orientations, suggesting structural stability compatible with the proposed inactive conformation. Although these trajectories do not capture long-timescale dynamics, multiple replicates enhance confidence in the observed arrangement. It should be noted that these short simulations primarily support the plausibility and stability of the observed interdomain arrangements, rather than providing exhaustive sampling of the kinase’s conformational space. Future studies with longer trajectories could more fully explore dynamic flexibility.

The functional relevance of the predicted pockets was probed using representative ligands. In WT breast cancer cells, both ligands reduced viability, suggesting that the interdomain pockets predicted in the full-length model are biologically accessible. Differential effects in overexpression systems highlight context-dependent accessibility: ligand 1, engaging a pocket directly at the C2/V5 interdomain space, showed enhanced cytotoxicity in C2-overexpressing cells, whereas ligand 2, targeting the PTD, modulated viability consistent with interference in phosphotyrosine-dependent interactions. Similarly, while ligands were used as functional probes, potential off-target effects in the cellular context cannot be excluded. Ligand 2 engages the PTD, a domain whose recognition motifs, unlike those of the homologous PKC-θ, utilise an aromatic context to bind phosphorylated tyrosines [32]. Ligand 1 binds a novel pocket at the C2/V5 interdomain space, providing functional evidence that this structurally uncharacterised region is accessible in full-length PKC-δ. Importantly, the primary goal of these experiments was to validate the structural accessibility and functional relevance of the predicted interdomain pockets, rather than to assess ligand selectivity or pharmacological potential. The consistent phenotypic outcomes observed across assays support the biological plausibility of the consensus model.

Overall, our consensus PKC-δ model demonstrates reproducible interdomain interactions, biologically plausible inactivity markers, and functional pocket accessibility. The Tyr64-dependent specificity of the C2/V5 interdomain space highlights opportunities for isoform-selective targeting, while integration of structural predictions with cell-based validation links computational insights to measurable phenotypic outcomes. Together, these results provide a predictive, functionally validated framework for domain-specific modulation of kinase activity.

## 4. Methods

### 4.1. Protein Sequence and Template Selection

The full-length human PKC-δ sequence was retrieved from NCBI RefSeq [33]. Structural templates for individual domains were identified from the Protein Data Bank (PDB) [34], prioritising high sequence identity and coverage, as well as the presence of conserved inactivity markers characteristic of PKC/AGC kinases [6,7,14,15]. Multiple sequence alignment was generated using COBALT (NCBI, Bethesda, MD, USA) [35]. Selected templates included the C1 and C2 domains (PDB: 1BDY [4], 1YRK [5], 2YUU [8], and 3UGD [36]), homologous kinase domains (1XJD [37]), and templates with structural features such as the pseudosubstrate occupying the substrate-binding site (6E9L [38] and 1ATP [22]), the C1b domain clamping the V5 domain (3PFQ [6]), displacement of the NFD motif from the ATP-binding site (6E9L), and tethering of lipophilic C1b residues to the catalytic domain (3PFQ). Templates lacking these markers were excluded. For more technical details, see Supplementary Materials Section S1, Table S1, and Figures S1 and S2.

### 4.2. Comparative Modelling and Refinement

Multi-template comparative modelling was performed with MODELLER v9.23 and v10.3 (Sali Lab, University of California, San Francisco, CA, USA) [39]. Twenty-four template order combinations were tested, generating ~6000 models across all runs. Models were visually inspected using UCSF Chimera v1.11, 1.13c, and 1.15c (University of California, San Francisco, CA, USA) [40], and evaluated with stereochemical validation tools, mainly MolProbity (Duke University, Durham, NC, USA) [41]. Those lacking one or more inactivity markers were discarded. A consensus model was constructed by integrating the most favourable features, with flexible loops predicted to form α-helices remodelled [42] (see Supplementary Materials Section S2). Zinc-binding sites were included for structural completeness. Iterative refinement was applied to the consensus model, using a combination of ModRefiner (Zhang Lab, University of Michigan, Ann Arbor, MI, USA) [43], GalaxyRefine (Center for Systems Biology, Seoul, Republic of Korea) [44], and MODELLER to reduce steric clashes, improve Ramachandran statistics, and optimise stereochemistry, with quality assessed at each step using MolProbity. Further details of the refinement workflow are provided in the Supplementary Materials Section S3.

### 4.3. Molecular Dynamics Simulations

The consensus full-length PKC-δ model was subjected to short all-atom MD simulations in explicit water with 0.15 M KCl using the CHARMM36m force field, prepared via CHARMM-GUI Solution Builder (University of Illinois at Urbana-Champaign, Urbana, IL, USA) [45], and simulated in OpenMM v8.3.1 (Stanford University, Stanford, CA, USA) [46]. Ten independent 5 ns simulations were performed at 310.15 K to assess the local stability of the fold and the interdomain interfaces. Trajectories were analysed using VMD (University of Illinois at Urbana-Champaign, Urbana, IL, USA) [17]. These simulations were intended for structural validation rather than extensive conformational sampling. Very soft positional restraints were applied to two highly flexible loops to maintain structural integrity, based on observations from longer exploratory simulations (100–300 ns; see Supplementary Materials Section S4 and Figure S3 for details).

### 4.4. Pocket Detection and Ligand Screening

Binding pockets in the C2 domain were first identified using DOGSiteScorer (BioSolveIT GmbH, Sankt Augustin, Germany) [19]. Four compound libraries were used: Asinex BioDiscovery, Asinex Lead-Like (both sourced from ASINEX Biodesign and Synergy/Lead-like collections), ZINC15 drug-like [47], and the University of Nottingham 2D compound library. Compounds were converted to 3D using LigPrep release 2020 (Schrödinger Inc., New York, NY, USA), generating nearly 1 million ligands divided into sublibraries for parallel docking.

Each sublibrary was docked against two target structures: the C2 domain isolated from our consensus model, and the crystallised C2 domain (PDB: 1BDY, chain B), using Glide release 2020 (Schrödinger Inc., New York, NY, USA) [18] through a tiered funnel of an initial SP screen, followed by XP redocking of the top poses. For each structure, the 30 highest-scoring and chemically tractable ligands were advanced and subsequently redocked into the complete C2 domain of the model, with an unrestricted grid. Final candidates were prioritised based on docking score, binding mode, and commercial availability. Docking results were analysed in Maestro release 2020-4 (Schrödinger Inc., New York, NY, USA). Full methodological details, pockets, and triage criteria are provided in the Supplementary Materials Section S5.

### 4.5. Cell Culture and Viability Assays

Human breast cancer cell lines (MCF-7, MDA-MB-468, MDA-MB-231) were cultured in Minimum Essential Medium Eagle supplemented with 10% foetal bovine serum, 1% L-glutamine, and 1% penicillin-streptomycin under standard conditions (37 °C, 5% CO_2_).

For this study, previously established MCF-7 cell lines stably expressing the isolated PKC-δ C2 domain (δC2 cells) or empty vector (Vector cells) were used. These cells were generated and validated in prior work [21].

Cell viability was assessed using MTT assays following treatment with candidate ligands alone or in combination with H_2_O_2_ for 48–72 h. Positive controls included etoposide (100 μM) and H_2_O_2_ (100 nM). Full experimental details and reagent sources are provided in the Supplementary Materials Section S6.

### 4.6. Statistical Analysis

Data distribution was assessed using Shapiro-Wilk normality tests (α = 0.05). Normally distributed data (*p* > 0.05) were analysed using one-way ANOVA with Dunnett’s multiple comparisons test for untransfected cell experiments, or two-way ANOVA with Tukey’s multiple comparisons for transfected cell experiments. All analyses were performed using GraphPad Prism 8 (GraphPad Software, San Diego, CA, USA) with statistical significance set at *p* < 0.05.

## 5. Conclusions

In summary, we present a full-length, inactive-state model of PKC-δ that captures key interdomain interactions and regulatory features. Functional validation with representative ligands confirms the biological relevance of predicted pockets and the utility of full-length modelling for identifying novel interdomain sites. This integrative approach provides mechanistic insights into PKC-δ regulation and offers a framework for future selective targeting of PKC isoforms or other AGC kinases.

## Data Availability

The raw data supporting the conclusions of this article will be made available by the authors on request.

## Supplementary Materials

The following supporting information can be downloaded at: https://www.mdpi.com/article/10.3390/ph18111760/s1. Supplementary Materials.docx provides detailed computational methods, additional results, and protocols for PKC-δ modelling, molecular dynamics simulations, and ligand docking/phenotypic validation. It includes four figures (Figures S1–S4) illustrating sequence alignment, template modelling, simulation analyses, and ligand docking, and five tables (Tables S1–S5) summarising template usage, model quality, druggable pockets, ligand properties, and ligand interactions. This Supplement provides sufficient detail to reproduce the computational and experimental procedures. Upon manuscript acceptance, the PKC-δ model will be deposited in a public repository (e.g., RCSB) with an accession code [4,5,6,8,17,18,19,20,21,22,32,33,34,35,36,37,38,39,40,41,42,43,44,45,46,47].
