# Supplementary material for: Modelling the Full-Length Inactive PKC-δ Structure to Explore Regulatory Accessibility and Selective Targeting Opportunities
Source: Pharmaceuticals (Basel). 2025 Nov 18;18(11):1760. doi: 10.3390/ph18111760 (PMC12655620; doi:10.3390/ph18111760)
Supplement: Supplementary file 1 [file pharmaceuticals-18-01760-s001.zip › pharmaceuticals-3963887-supplementary.pdf]

pharmaceuticals

MDPI

# Supplementary Materials: Modelling the Full-Length Inactive PKC-δ Structure to Explore Regulatory Accessibility and Selective Targeting Opportunities

**Rasha Khader and Lodewijk V. Dekker**

The Supplementary Materials provide detailed methodological and validation data for protein kinase C-δ (PKC-δ) modelling, molecular dynamics (MD) simulations, and ligand docking/biological validation studies. Tables include: **Table S1**, listing all structural templates used for multi-template modelling; **Table S2**, summarising model quality scores before and after refinement; **Table S3**, characteristics of druggable pockets; **Table S4**, a consolidated dataset of XP (2) docking scores, ligand properties, and normalised viability values; and **Table S5**, a summary of key ligand/C2 domain interactions and bond lengths**.** Figures include: **Figure S1**, a schematic of the 2D PKC-δ sequence alignment with template coverage; **Figure S2**, illustrating the MOD1 composite template used to model the C2/V3 domains interface; **Figure S3**, showing RMSD and structural changes during the initial 100 ns simulations before applying harmonic restraints; and **Figure S4**, pockets and comparing SP versus XP (2) docking poses of ligands in 3D. This Supplement provides sufficient detail for readers to reproduce the modelling and docking procedures and explains some of the structural features recognised during modelling. Upon manuscript acceptance, the model (as a PDB) will be deposited in a public repository (e.g., RCSB) with an accession code. This model represents a computationally derived structure and should be interpreted in the context of the applied homology modelling and phenotypic validation.

## Section S1: Protein sequence and template selection

The full-length sequence of human protein kinase C-δ (PKC-δ) used for comparative modelling was obtained from the NCBI RefSeq (Accession number: NP_006245.2) [33]. Structural templates for individual domains were identified from crystallised structures deposited in the RCSB Protein Data Bank (PDB) [34]. FASTA sequences were retrieved from NCBI, and the initial alignment was generated using COBALT [35].

Since not all domains of PKC-δ have been crystallised, homologous structures from closely related kinases were employed. Templates were first filtered based on sequence identity and coverage, then prioritised according to the presence of conserved inactivity markers characteristic of this kinase family. These markers, which correlate with conformational switching and domain regulation, ensured that selected templates could reproduce biologically relevant inactive domain orientations. Templates lacking motifs critical for building the inactive conformation were excluded from downstream multi-template modelling. A complete list of templates and their provided structural elements is shown in Table S1.
Key motifs considered include:

- the pseudosubstrate binding region within the substrate-binding domain [22, 38],
- the C1b subdomain clamping the V5 domain [6],
- the AGC family conserved NFD sequence adopting a helix-out conformation away from the ATP-binding site [6, 38],
- and the C1b subdomain membrane-binding loop tethered to the catalytic domain [6].

A simplified alignment of the kinase and templates is presented in Figure S1.

**Table S1.** Structural templates used in this study, the features they provided, and their kinase/species of origin.

| PDB-chain | Structural features | Species | Kinase | Reference |
|---|---|---|---|---|
| **1BDY-B** | The isolated C2 domain. | Human | PKC-δ | [4] |
| **1YRK-A/B** | The C2/V3 domains interaction. | Rat | PKC-δ | [5] |
| **2YUU-A** | The C1a $Zn^{2+}$-binding loops. The diacylglycerol/phorbol esters-binding site. | Human | PKC-δ | [8] |
| **3UGD-A** | The C1b $Zn^{2+}$-binding loops. The diacylglycerol/phorbol esters-binding site. | Mouse | PKC-δ | [36] |
| **3PFQ-A*** | The C1b/catalytic domains interaction. The C1b domain clamp of the V5 domain. The inactive NFD folded outside the ATP-binding site. The C-terminal of the V5 domain positioned between the inactive αC-helix and β4-strand in the C3 domain. The template with the most completed V5 domain. | Rat | PKC-βII | [6] |
| **1XJD-A** | The catalytic domain from the isoform most homologous to PKC-δ. Structure for the substrate-and ATP-binding sites. | Human | PKC-θ | [37] |
| **1ATP-E/I** | Pseudosubstrate interactions with the substrate-binding site, with focus on the ATP-binding site. | Mouse | PKA | [22] |
| **6E9L-A/B** | Pseudosubstrate interactions with the substrate-binding site, with focus on the substrate-binding site. | Human | PKA | [38] |

*PKC-βII C2 domain was excluded from the PDB.

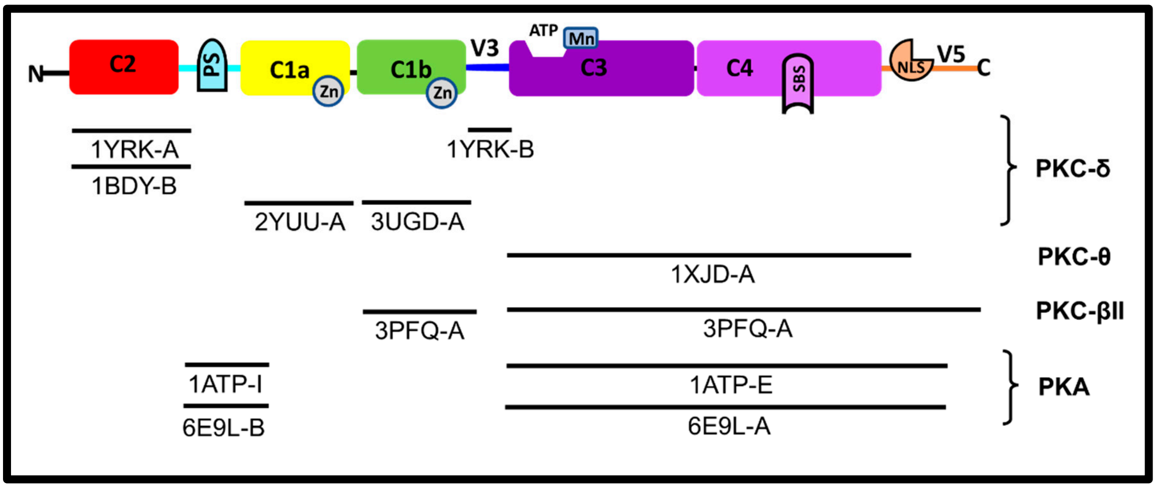

**Figure S1.** Simplified alignment. Templates are aligned with the corresponding domains in the 2D structure of PKC-δ, and the AGC kinase from which each template was derived is indicated.

## Section S2: Comparative modelling

Multi-template modelling was performed using MODELLER 9.23 and 10.3 [39]. To minimise cross-template conflicts and to evaluate the contribution of each template, modelling was initiated with one template per domain, and the number of templates per domain was then gradually increased in a domain-by-domain manner.

We observed that the resulting models varied depending on the order in which template blocks were introduced. MODELLER treats templates within the same block as interchangeable, but the order of the blocks themselves was critical. The template blocks were defined as follows:

Block A: 1BDY and 1YRK.
Block B: 2YUU.
Block C: 3UGD and 3PFQ.
Block D: 1ATP, 6E9L, 1XJD.

The initial trial included one template from each block (1BDY, 2YUU, 3PFQ, 1ATP). In subsequent trials, additional templates were introduced block by block to examine the structural contribution of each. For each trial, all possible block orders (4! = 24 permutations) were tested, and each run generated 10–20 replicate models. In total, >4000 models were initially created.

Certain trials produced open/active-like conformations of the kinase; these consistently arose when 1YRK chain B, 1ATP chain I and/or 6E9L chain B, or 3PFQ were excluded. Models from these trials were excluded. Similarly, runs starting with Block B often resulted in misfolded domains and were discarded.
We also observed that adding too many templates per domain sometimes overwhelmed MODELLER, producing steric clashes or domain intercalations. For example:

- Including all Block D templates caused the V5 domain to intercalate into the catalytic domain; exclusion of 1ATP resolved this.
- Using both 1BDY and 1YRK in Block A led to intercalation between the C2 and catalytic domains.
- Using only 1YRK in Block A caused clashes near Gly99.

While many models were discarded, inclusion of 1YRK was essential for correctly positioning the V3 domain relative to the C2 domain. Functional studies have shown that phosphorylated Tyr313 within V3 interacts with the phosphotyrosine-binding region of the C2 domain to activate PKC-δ [20, 32], supporting their close spatial proximity. Indeed, when we attempted to model the kinase without enforcing this C2/V3 interface, the resulting structures consistently adopted open/active-like conformations. To preserve an inactive architecture while capturing this interaction, we pre-modelled the C2 and V3 domains together in MODELLER using both 1BDY and 1YRK, generating a composite template (''MOD1''). MOD1 (Figure S2) was then used in place of Block A in subsequent trials, increasing the total dataset to >6000 models.

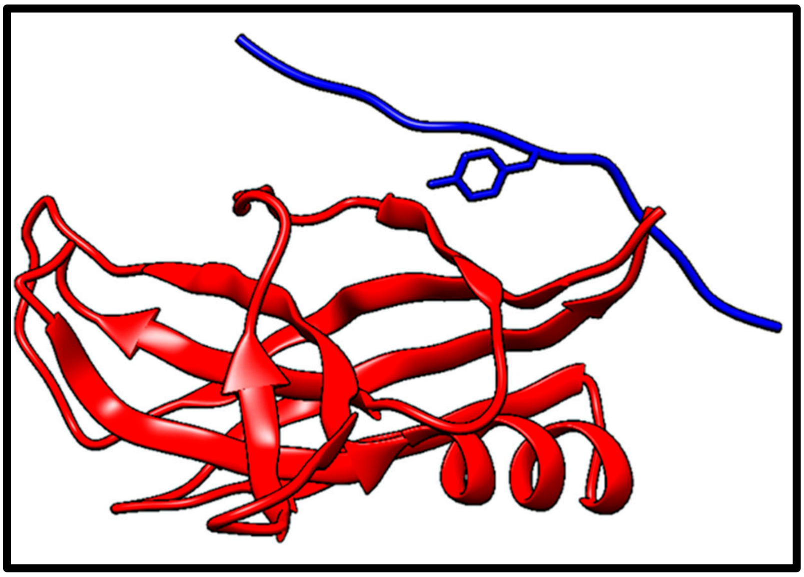

**Figure S2.** Template MOD1. Composite model of the C2 (red) and V3 (blue) domains generated from 1BDY chain B and 1YRK chains A and B. The V3 segment (Ser307–Asp326) is positioned adjacent to the C2 domain, consistent with reported phosphorylated Tyr313 interactions. Tyr313 is highlighted for reference.

Models were filtered visually after each run using Chimera 1.11, 1.13c, and 1.15c [40], and candidate inactive conformations were scored in MolProbity (>1000 models) [41]. Models with optimisable stereochemistry (Ramachandran favoured >85%, clashscore <160, Cβ deviation <5 Å) were grouped and re-inspected feature by feature. Since the remaining models were broadly comparable, the 10 highest-scoring models were selected and used as templates to generate the final consensus model.

## Section S3: Consensus model loops and refinement

The consensus model contained three highly flexible loops: Gly210–Phe225, Ala290–Ser306, and Phe665–Asp676, of which the latter two lacked template coverage. Loop conformations were predicted using PEP-FOLD3 [42], which suggested that residues Ser215–Arg224 and Leu291–Ser302 adopt $\alpha$-helical structures. Accordingly, the consensus model was remodelled in MODELLER, applying $\alpha$-helix restraints for these loops, with 20 replicate models generated per loop.

The model was then subjected to a multi-step refinement protocol:

1. ModRefiner [43]: optimised Ramachandran statistics and Cβ deviations.
2. GalaxyRefine [44]: reduced clashscores to values acceptable by MODELLER.
3. MODELLER: applied final adjustments to clashscores, Ramachandran statistics, and Cβ deviations.

Refinement scores before and after optimisation are reported in Table S2.

For completeness, four zinc-binding sites (Zn1–Zn4) were included, though they were not the focus of this study. The sites were manually adjusted in Chimera and did not affect the final stereochemical scores. One cysteine ligand per site displayed suboptimal coordination; however, this did not majorly disrupt the overall fold or domain orientations during subsequent simulations.

**Table S2.** Model scores before and after refinement.

| Score | Before | After |
|---|---|---|
| **Clashscore** | 150.65 (0$^{th}$ percentile) | 0 (100$^{th}$ percentile) |
| **Ramachandran favoured** | 92.14% | 96.44% |

| | | |
|---|---|---|
| **Ramachandran outliers** | 2.37% | 1.19% |
| **Ramachandran distribution Z-score** | -1.04 ± 0.29 | 0.22 ± 0.3 |
| **MolProbity score** | 3.65 (6th percentile) | 0.94 (100th percentile) |
| **Cβ deviations > 0.25Å** | 3.96% | 1.58% |
| **Bad bonds** | 0.97% | 0 |
| **Bad angles** | 2.8% | 1.43% |

## Section S4: Molecular dynamics simulations

The consensus model was prepared for molecular dynamics (MD) simulations using CHARMM-GUI Solution Builder [45] with the CHARMM36m force field, at pH 7.4, 310.15 K, in a 0.15 M KCl aqueous box under NVT conditions. The kinase was placed in a cubic water box with a minimum distance of 12.7 nm from the protein surface to the box edges, ensuring that no interactions occurred between periodic images. Simulations were performed using OpenMM v8.3.1 [46]. Initial production runs were performed for 100–300 ns, during which we observed early movement of the V3 domain. This movement led to the gradual activation of the kinase and loss of inactivity markers, supporting our previous hypothesis that the C2 and V3 domains are in close proximity in the inactive kinase. The observed shift of the V3 domain toward an active conformation is expected, as the template used to construct the model was derived from the active state. In simulations beyond 100 ns, the V5 loop (residues Phe660–Asp676) began to fold onto itself, causing the RMSD to increase sharply to over 15 Å. It should be noted that the terminal portion of the V5 domain corresponded to an alignment loop for which no structural template was available.

To stabilise the inactive conformation, we applied soft harmonic restraints (50 kJ/mol/nm$^2$) to the backbone atoms of the V3 domain (residues Leu291–Thr333) and the C-terminal portion of the V5 domain (residues Phe660–Asp676). Ten independent replicate simulations of 5 ns each were then performed. Longer simulations were not feasible due to computational limitations at this stage. Results were analysed using VMD [17], and the RMSD and overall structural behaviour for the 100 ns run are shown in Figure S3.

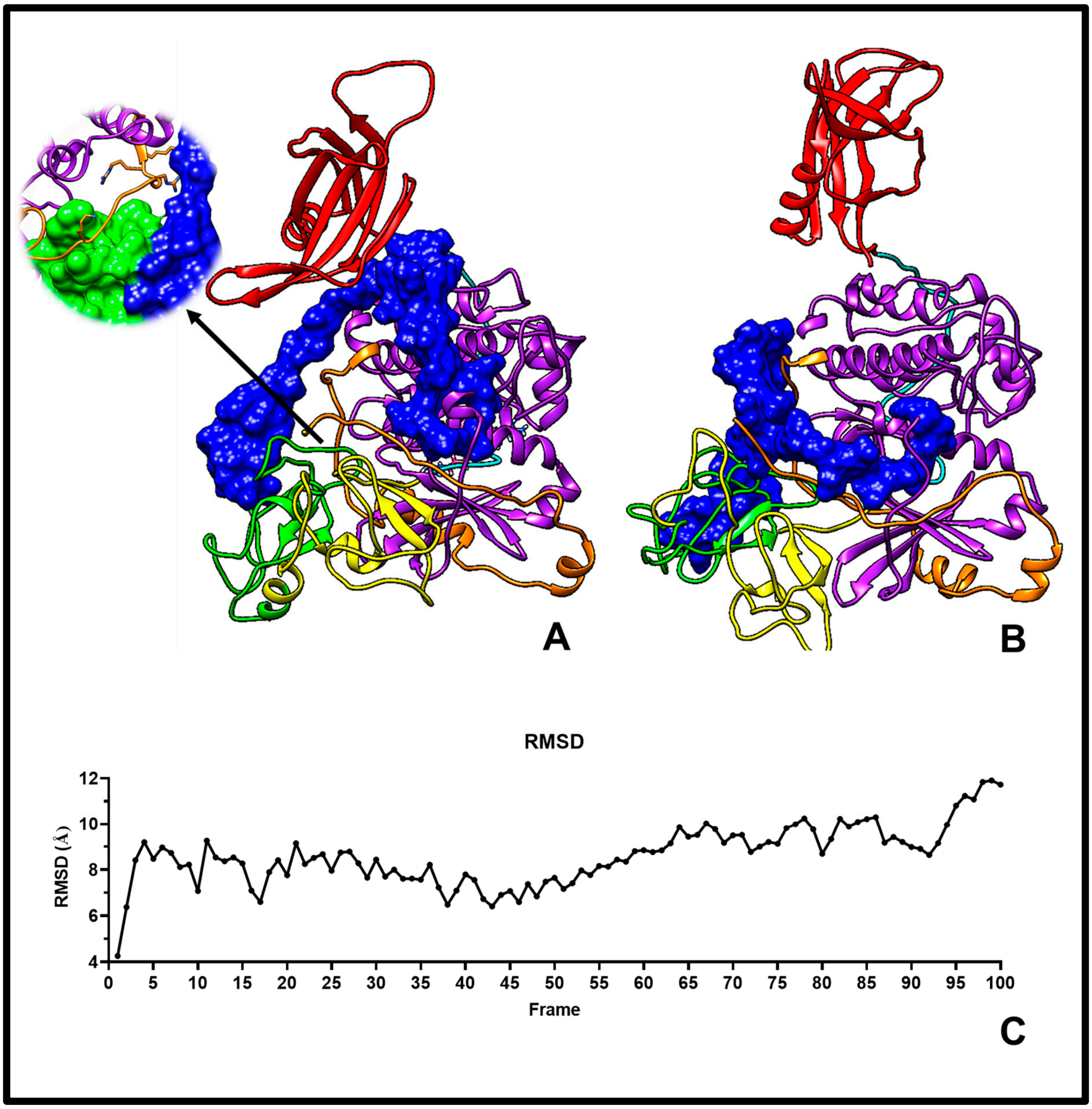


**Figure S3.** RMSD and structural dynamics during the 100 ns simulation. **(A)** The starting structure, with the V3 domain (blue) positioned near the C2 domain (red). **(B)** After 100 ns of simulations, the V3 domain drifts away, inducing rotational rearrangements in the C2 domain and its displacement from both the catalytic domain (purple) and the V5 domain (orange). This motion stretches the pseudosubstrate segment (cyan), suggesting an early detachment from the substrate-binding site. Concurrently, the C1a subdomain (yellow) begins to disengage from the catalytic domain, while the C1b subdomain (green) loosens its clamp over the V5 domain (the clamp region is highlighted in panel A). The V5 domain also starts to detach, most prominently around the NF$^{633}$D motif. **(C)** RMSD plot for the 100 ns trajectory (1 frame/ns) prior to applying restraints to the V3 domain (residues Leu291–Thr333). The RMSD rises sharply to ~8–9 Å by 5 ns (frame 5), indicating early large-scale conformational changes.

## Section S5: Pocket detection and ligand screening

The isolated C2 domain from the consensus model (tan, Figure S4) and the C2 domain crystal (1BDY chain B, silver, Figure S4) were analysed for putative binding pockets using ProteinsPlus DOGSiteScorer [19]. Six druggable pockets were identified in both structures (Figure S4A, B). On the face oriented toward the interdomain space with the V5 domain, three pockets were identified. The initial docking grid (black box, Figure S4A, B) for each structure was therefore defined by these pockets, although none represented the top-ranked pocket in either structure. Pocket characteristics are summarised in Table S3.

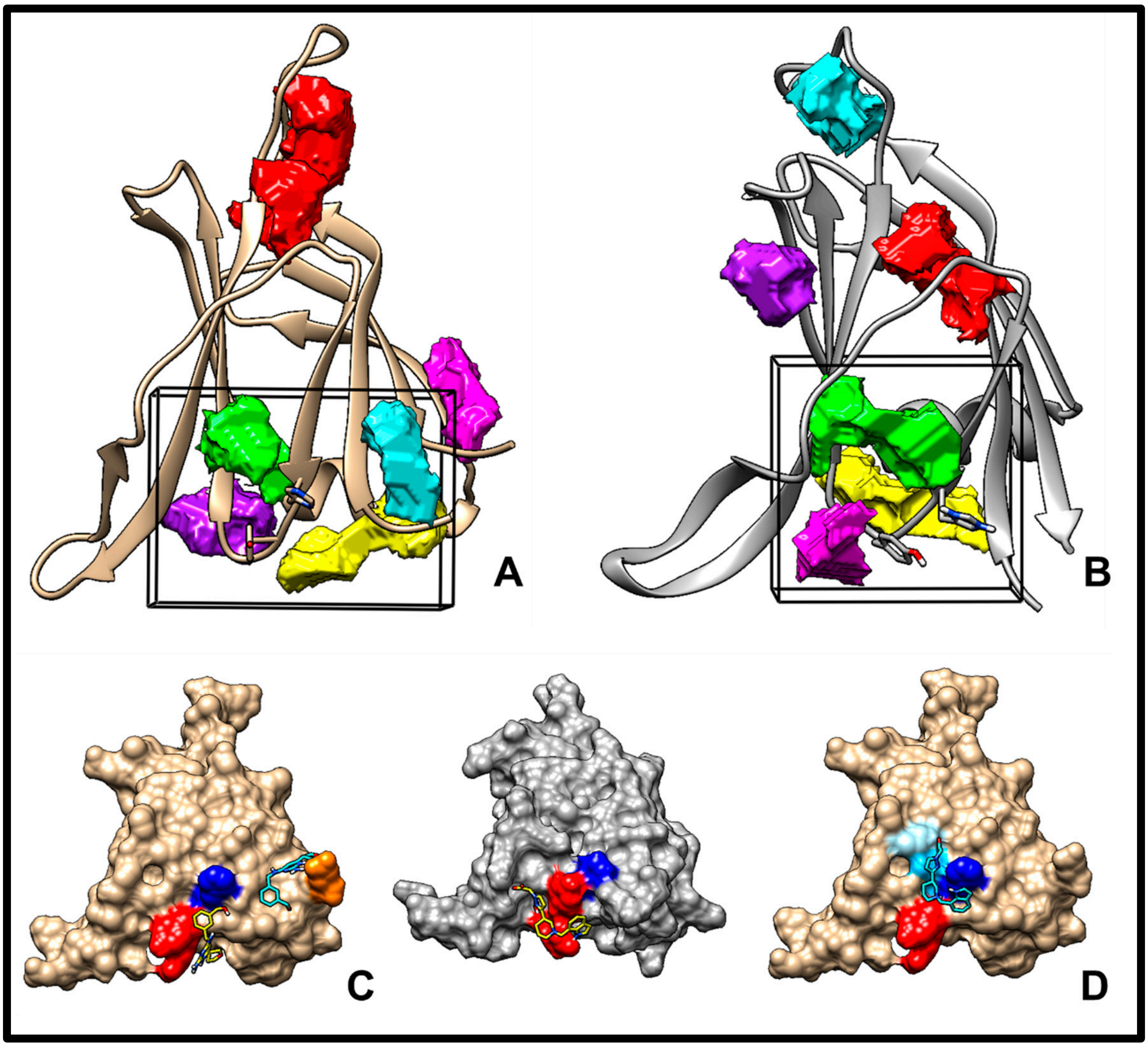


**Figure S4.** Pockets, docking grids, and ligand binding. Druggable pockets in the **(A)** C2 domain of the model compared with the **(B)** C2 domain of 1BDY chain B, ranked by druggability (red > yellow > green > cyan > purple > magenta). The initial docking grid (black box) encompassed all pockets on the face oriented toward the V5 domain. Tyr64 and His62 are shown for reference. **(C)** Binding pocket/mode of ligand 1 after initial SP docking (yellow) and after XP (2) redocking (cyan). Key residues: Tyr64 and Glu65 (red), His62 (blue), Glu123 (orange). **(D)** Binding pocket of ligand 2 after initial SP docking (yellow) in 1BDY chain B (silver) compared with the XP (2) run (cyan) in the full C2 domain from the model (tan). Tyr64 and Glu65 are shown (red); the phosphotyrosine-binding site is highlighted in blue shades.

**Table S3.** Characteristics of druggable pockets in the C2 domain from the model vs in 1BDY chain B. Pockets are named according to their colours in Figure S4.

| Structure | Feature | Red | Yellow | Green | Cyan | Purple | Magenta |
|---|---|---|---|---|---|---|---|
| C2 domain from the model | **Druggability score** | 0.64 | 0.51 | 0.38 | 0.24 | 0.24 | 0.28 |
| | **Volume (Å³)** | 425.5 | 166.7 | 149.3 | 116.9 | 106.3 | 104.7 |
| | **Hydrophobicity** | 0.25 | 0.44 | 0.31 | 0.48 | 0.42 | 0.43 |
| | **Hydrogen bond acceptors** | 42 | 19 | 8 | 14 | 12 | 18 |
| | **Hydrogen bond donors** | 14 | 6 | 10 | 2 | 6 | 5 |
| 1BDY chain B | **Druggability score** | 0.45 | 0.31 | 0.38 | 0.35 | 0.22 | 0.28 |
| | **Volume (Å³)** | 169.1 | 165.7 | 151.8 | 148.2 | 148.1 | 124.8 |

| | | | | | | |
|---|---|---|---|---|---|---|
| **Hydrophobicity** | 0.38 | 0.33 | 0.35 | 0.35 | 0.46 | 0.6 |
| **Hydrogen bond acceptors** | 20 | 18 | 16 | 19 | 9 | 12 |
| **Hydrogen bond donors** | 6 | 8 | 12 | 9 | 6 | 5 |

Two-dimensional ligands from the Asinex BioDiscovery and Lead-Like libraries (both sourced from ASINEX Biodesign and Synergy/Lead-like collections), ZINC15 drug-like library [47], and the University of Nottingham 2D Compound Library were converted to 3D using LigPrep release 2020. Approximately one million compounds were generated and divided into sublibraries of 100,000 compounds.

Each sublibrary was docked in parallel into (i) the C2 domain from the consensus model and (ii) the isolated C2 domain crystal (1BDY chain B) using Glide SP [18], with three poses per ligand (20 SP runs in total). The top 30 ligands/poses from each run (300 ligands per structure) were then redocked using Glide XP into the same initial grid (two XP (1) runs total).
From this pool, the top 30 ligands per structure were docked a third time into the entire C2 domain of the model, with no predefined pocket, using Glide XP (XP (2) run). Ligands were triaged at each stage according to docking score, size, and binding interactions, with commercial availability prioritised at the final stage. Two candidates were selected: Ligand 1 from the model-derived pool and Ligand 2 from the 1BDY chain B-derived pool.

Ligand 1 (Figure S4C) was initially docked near Tyr64 in the SP run, occupying the yellow pocket (Figure S4A; yellow pose in Figure S4C). After XP (2) docking into the full C2 domain, the preferred pose shifted towards the cyan pocket (Figure S4A; cyan pose in Figure S4C). Both pockets are located at the C2/V5 interdomain surface, suggesting that such ligands could potentially interfere with C2/V5 reassociation after activation.

Ligand 2 (Figure S4D) was likewise initially placed near Tyr64 during SP docking (magenta pocket Figure S4B; yellow pose in Figure S4D). Following XP (2) docking into the full C2 domain, it relocated upward toward the green pocket (Figure S4B; cyan pose in Figure S4D), which corresponds to the established phosphotyrosine-binding site of the C2 domain [32].

A consolidated dataset including SP/XP docking scores, key ligand properties, and normalised viability values for both wild-type and δC2-overexpressing MCF-7 cells is presented in Table S4, while, a summary of ligand/C2 domain interactions, including hydrogen bonds, salt bridges, π–cation, and ring-stacking interactions, along with measured bond lengths, enabling comparison with cellular activity, is presented in Table 5.

**Table S4.** Docking scores, properties, and viability data of the two ligands.

| Feature | Ligand 1 | Ligand 2 |
|---|---|---|
| **ZINC ID** | ZINC299769510 | ZINC426477475 |
| **Purchased from** | ChemBridge, San Diego, USA, through Hit2Lead | |
| **Purity guaranteed from the provider** | 99% | 99% |
| **SP docking score (kcal/mol)** | -5.8 | -4.8 |
| **XP (2) docking score (in the full C2 domain, kcal/mol)** | -6.5 | -5.5 |
| **Molecular weight (g/mol)** | 300.362 | 338.411 |
| **LogP** | 0.71 | 1.76 |
| **Net charge** | +1 | 0 |
| **Hydrogen bond donors** | 3 | 2 |
| **Hydrogen bond acceptors** | 5 | 4 |
| **Rotatable bonds** | 5 | 5 |
| **Mean normalised viability in wild type cells (%)** | | |
| **MCF-7** | 44.6 | 28.8 |

| | | |
|---|---|---|
| **MDA-MB-468** | 54.6 | 36.0 |
| **MDA-MB-231** | 48.7 | 36.9 |
| **Mean normalised viability in transfected MCF-7 cells (%)** | | |
| **Single treatment: δC2-overexpressing** | 28.3 | 52.0 |
| **Single treatment: Vector control** | 65.5 | 18.4 |
| **Co-treatment with $H_2O_2$: δC2-overexpressing** | 49.3 | 65.8 |
| **Co-treatment with $H_2O_2$: Vector control** | 49.3 | 27.0 |

**Table S5.** Key ligand/C2 domain interactions and bond lengths for both ligands from XP (2) docking.

| Ligand | Protein Residue | Interaction Type | Distance (Å) |
|---|---|---|---|
| **Ligand 1** | **Glu123** | **H-bond (NH)** | **2.01** |
| | **Glu123** | **H-bond (NH2)** | **1.73** |
| | **Glu123** | **Salt bridge** | **4.73** |
| | **Met1** | **H-bond (OH)** | **2.10** |
| | **Phe121** | **H-bond (OH)** | **1.85** |
| **Ligand 2** | **Lys48** | **H-bond (carboxylic O)** | **1.83** |
| | **Lys48** | **Salt bridge** | **3.52** |
| | **Arg67** | **Salt bridge** | **3.52** |
| | **Arg67** | **Pi–cation (pyrazole)** | **3.82** |
| | **His62** | **Ring stacking (benzopyrrole)** | **3.70** |

## Section S6: Cell culture and viability assays

Human breast cancer cell lines MCF-7, MDA-MB-468, and MDA-MB-231 were cultured in Minimum Essential Medium Eagle (Sigma-Aldrich, Gillingham, UK) supplemented with 10% foetal bovine serum (Sigma-Aldrich, Gillingham, UK), 1% L-glutamine (200 mM; Gibco, São Paulo, Brazil), and 1% penicillin-streptomycin (Gibco, São Paulo, Brazil) at 37°C and 5% $CO_2$.

For this study, previously established MCF-7 cell lines stably overexpressing the isolated PKC-δ C2 domain (δC2 cells) or empty vector (vector cells) were used as provided [21]. These cells were used without further modification for all experiments described here.

Cell viability was assessed using MTT assays following treatment with ligand 1 or 2 alone (100 μM) or in combination with $H_2O_2$ (100 nM). Etoposide (100 μM) and $H_2O_2$ (100 nM) were included as positive controls. Cells were seeded at $4 \times 10^3$ cells/well in 96-well plates and incubated for 24 h before treatment, followed by an additional 48-72-h incubation.

For MTT measurement, 50 μL of 0.2% (w/v) thiazolyl blue tetrazolium bromide in PBS was added to each well. Plates were incubated for 3 h, after which the solution was aspirated and replaced with 50 μL of 99.99% DMSO. Plates were shaken in the dark for 1 minute, and absorbance was measured at 570 nm. Readings were normalised to the viability of untreated controls.

## References


4. Pappa, H.; Murray-Rust, J.; Dekker, L.V.; Parker, P.J.; McDonald, N.Q. Crystal Structure of the C2 Domain from Protein Kinase C-Delta. *Structure* **1998**, 6, 885–894.
5. Sondermann, H.; Kuriyan, J. C2 Can Do It, Too. *Cell* **2005**, 121, 158–160.

6. Leonard, T.A.; Różycki, B.; Saidi, L.F.; Hummer, G.; Hurley, J.H. Crystal Structure and Allosteric Activation of Protein Kinase C Bii. *Cell* **2011**, 144, 55–66.
8. Abe, H.; Miyamoto, K.; Tochio, N.; Saito, K.; Sasagawa, A.; Koshiba, S.; Inoue, M.; Kigawa, T.; Yokoyama, S. Solution Structure of the First Phorbol Esters/Diacylglycerol Binding Domain of Human Protein Kinase C, Delta. *Protein Data Bank* **2008**, *To be published*.
17. Humphrey, W.; Dalke, A.; Schulten, K. Vmd - Visual Molecular Dynamics. *J. Molec. Graph.* **1996**, 14, 33–38.
18. Friesner, R.A.; Banks, J.L.; Murphy, R.B.; Halgren, T.A.; Klicic, J.J.; Mainz, D.T.; Repasky, M.P.; Knoll, E.H.; Perry, J.K.; Shaw, D.E.; et al. Glide: A New Approach for Rapid, Accurate Docking and Scoring. 1. Method and Assessment of Docking Accuracy. *J. Med. Chem.* **2004**, 47, 1739–1749.
19. Volkamer, A.; Kuhn, D.; Rippmann, F.; Rarey, M. Dogsitescorer: A Web Server for Automatic Binding Site Prediction, Analysis and Druggability Assessment. *Bioinformatics* **2012**, 28, 2074–2075. Available online at: https://proteins.plus/ (last accessed 25 September 2025).
20. Lu, W.; Finnis, S.; Xiang, C.; Lee, H.K.; Markowitz, Y.; Okhrimenko, H.; Brodie, C. Tyrosine 311 Is Phosphorylated by C-Abl and Promotes the Apoptotic Effect of Pkcδ in Glioma Cells. *Biochem. Biophys. Res. Commun.* **2006**, 352, 431–436.
21. Khader, R.; Dekker, L.V. The C2 Domain of Pkc-Δ as a Dominant-Negative Modulator of Breast Cancer Cell Survival and Chemosensitivity. *ACS Omega* **2025**, Manuscript submitted.
22. Zheng, J.; Trafny, E.A.; Knighton, D.R.; Xuong, N.; Taylor, S.S.; Ten Eyck, L.F.; Sowadski, J.M. 2.2 Å Refined Crystal Structure of the Catalytic Subunit of Camp-Dependent Protein Kinase Complexed with Mnatp and a Peptide Inhibitor. *Acta Crystallogr. D Biol. Crystallogr.* **1993**, 49, 362–365.
32. Benes, C.H.; Wu, N.; Elia, A.E.H.; Dharia, T.; Cantley, L.C.; Soltoff, S.P. The C2 Domain of Pkcdelta Is a Phosphotyrosine Binding Domain. *Cell* **2005**, 121, 271–280.
33. O'Leary, N.A.; Wright, M.W.; Brister, J.R.; Ciufo, S.; Haddad, D.; McVeigh, R.; Rajput, B.; Robbertse, B.; Smith-White, B.; Ako-Adjei, D.; et al. Reference Sequence (Refseq) Database at Ncbi: Current Status, Taxonomic Expansion, and Functional Annotation. *Nucleic Acids Res.* **2016**, 44, D733–D745. Available online at: https://www.ncbi.nlm.nih.gov/refseq/ (last accessed 30 March 2025).
34. Burley, S.K.; Bhatt, R.; Bhikadiya, C.; Bi, C.; Biester, S.; Biswas, P.; Bittrich, S.; Blaumann, S.; Brown, R.; Chao, H.; et al. Updated Resources for Exploring Experimentally-Determined Pdb Structures and Computed Structure Models at the Rcsb Protein Data Bank. *Nucleic Acids Res.* **2025**, 53, D564–D574. Available online at: https://www.rcsb.org/ (last accessed 1 October 2025).
35. Papadopoulos, J.S.; Agarwala, R. Cobalt: Constraint-Based Alignment Tool for Multiple Protein Sequences. *Bioinformatics* **2007**, 23, 1073–1079. Available online at: https://www.ncbi.nlm.nih.gov/tools/cobalt/re_cobalt.cgi (last accessed 1 March 2025).
36. Shanmugasundararaj, S.; Das, J.; Sandberg, W.S.; Zhou, X.; Wand, D.; Messing, R.O.; Bruzik, K.S.; Stehle, T.; Miller, K.W. Structural and Functional Characterization of an Anesthetic Binding Site in the Second Cysteine-Rich Domain of Protein Kinase Cdelta. *Biophys. J.* **2012**, 103, 2331–2340.
37. Xu, Z.B.; Chaudhary, D.; Olland, S.; Wolfrom, S.; Czerwinski, R.; Malakain, K.; Lin, L.; Stahl, M.L.; Joseph-McCarthy, D.; Benander, C.; et al. Catalytic Domain Crystal Structure of Protein Kinase C-Theta (Pkctheta). *J. Biol. Chem.* **2004**, 279, 50401–50409.
38. Hobson, A.D.; Judge, R.A.; Aguirre, A.L.; Brown, B.S.; Cui, Y.; Ding, P.; Domingues, E.; DiGiammarino, E.; Egan, D.A.; Freiberg, G.M; et al. Identification of Selective Dual Rock1 and Rock2 Inhibitors Using Structure-Based Drug Design. *J. Med. Chem.* **2018**, 61, 11074–11100.
39. Sali, A.; Blundell, T.L. Comparative Protein Modelling by Satisfaction of Spatial Restraints. *J. Mol. Biol.* **1993**, 234, 779–815.
40. Pettersen, E.F.; Goddard, T.D.; Huang, C.C.; Couch, G.S.; Greenblatt, D.M.; Meng, E.C.; Ferrin, T.E. Ucsf Chimera--a Visualization System for Exploratory Research and Analysis. *J. Comput. Chem.* **2004**, 25, 1605–1612.
41. Chen, V.B.; Arendall, W.B. 3rd; Headd, J.J.; Keedy, D.A.; Immormino, R.M.; Kapral, G.J.; Murray, L.W.; Richardson, J.S.; Richardson, D.C. Molprobity: All-Atom Structure Validation for Macromolecular Crystallography. *Acta Crystallogr. D Biol. Crystallogr.* **2010**, 66, 12–21. Available online at: https://molprobity.biochem.duke.edu/ (last accessed 1 October 2025).
42. Lamiable, A.; Thévenet, P.; Rey, J.; Vavrusa, M.; Derreumaux, P.; Tufféry, P. Pep-Fold3: Faster De Novo Structure Prediction for Linear Peptides in Solution and in Complex. *Nucleic Acids Res.* **2016**, 44, W449–W454. Available online at: https://bioserv.rpbs.univ-paris-diderot.fr/services/PEP-FOLD3/ (last accessed 5 July 2025).

43. Xu, D.; Zhang, Y. Improving the Physical Realism and Structural Accuracy of Protein Models by a Two-Step Atomic-Level Energy Minimization. *Biophys. J.* **2011**, 101, 2525–2534. Available online at: https://www.aideepmed.com/ModRefiner/ (last accessed 30 July 2025).
44. Heo, L.; Park, H.; Seok, C. Galaxyrefine: Protein Structure Refinement Driven by Side-Chain Repacking. *Nucleic Acids Res.* **2013**, 41, W384–W388. Available online at: https://galaxy.seoklab.org/cgi-bin/submit.cgi?type=REFINE (last accessed 30 July 2025).
45. Jo, S.; Kim, T.; Iyer, V.G.; Im, W. Charmm-Gui: A Web-Based Graphical User Interface for Charmm. *J. Comput. Chem.* **2008**, 29, 1859–1865. Available online at: https://www.charmm-gui.org/ (last accessed 30 August 2025).
46. Eastman, P.; Friedrichs, M.S.; Chodera, J.D.; Radmer, R.J.; Bruns, C.M.; Ku, J.P.; Beauchamp, K.A.; Lane, T.J.; Wang, L.P.; Shukla, D.; et al. Openmm 4: A Reusable, Extensible, Hardware Independent Library for High Performance Molecular Simulation. *J. Chem. Theor. Comput.* **2013**, 9, 461–469.
47. Sterling, T.; Irwin, J.J. Zinc 15 – Ligand Discovery for Everyone. *J. Chem. Inf. Model.* **2015**, 55, 2324–2337.
